# NS‐*Pten* knockout mice show sex‐ and age‐specific differences in ultrasonic vocalizations

**DOI:** 10.1002/brb3.857

**Published:** 2017-10-18

**Authors:** Matthew S. Binder, Joaquin N. Lugo

**Affiliations:** ^1^ Department of Psychology and Neuroscience Baylor University Waco TX USA; ^2^ Department of Biology Baylor University Waco TX USA; ^3^ Institute of Biomedical Studies Baylor University Waco TX USA

**Keywords:** autism spectrum disorder, communication deficits, maternal isolation, *Pten*, sexually dimorphic, ultrasonic vocalization

## Abstract

**Objective:**

The goal of this study was to identify changes in quantitative and qualitative aspects of neonatal ultrasonic vocalizations USVs in neuron‐subset specific (NS‐*Pten)* knockout males and females when compared with wild‐type male and female mice.

**Background:**

One signaling cascade that plays a crucial role in the development of an autistic‐like phenotype is the PI3K/Akt/mTOR pathway. Mouse models that illustrate this connection include *Fmr1*,* Tsc1,* and NS‐*Pten*‐deficient mice. While numerous studies have investigated ultrasonic vocalizations in *Fmr1* knockout and *Tsc1* heterogenous mice, none have investigated USVs in NS‐*Pten* knockout mice using a full spectrum recording system.

**Methods:**

We recorded ultrasonic vocalizations from NS‐*Pten* wild‐type and knockout male and female mice on postnatal days 8 and 11. On these days, we measured the number and quality of calls emitted from pups when they were removed from their mothers.

**Results:**

We found that knockout pups emitted fewer vocalizations for both sexes (*p *<* *.05). Knockout males had calls of a shorter duration and lower peak amplitude on day 8, while showing a shorter duration, lower peak amplitude, and higher peak and fundamental frequency on day 11 (*p *<* *.001). Knockout females vocalized at a lower peak amplitude and fundamental frequency, and a higher peak frequency on day 8, while showing a shorter duration and a higher peak and fundamental frequency on day 11 (*p *<* *.001). Spectrographic analyses also revealed significant differences in call type for both genotypes and sexes (*p *<* *.05).

**Conclusions:**

These findings demonstrate that deletion of NS‐*Pten* results in significant decreases in vocalizations across both sexes. Additionally, our findings indicate that the aberrant vocalizations and increased call duration seen in other mTOR models are also present in NS‐*Pten* knockout mice. Our study provides evidence of a connection between hyperactive mTOR signaling and neonatal ultrasonic vocalizations.

## 1 INTRODUCTION

Autism spectrum disorder (ASD) is a neurodevelopmental condition that appears in early childhood and is characterized by impairments in social interaction, communication, and repetitive behaviors (Rapin & Tuchman, 2008). A recent survey by the Autism and Developmental Disabilities Monitoring Network (ADDM) estimated that the prevalence of ASD among 8‐year‐old children is 1 in 42 for boys and 1 in 189 for girls (Wingate et al., 2014). Since there is such a high concordance rate (70–90%) of monozygotic twins compared to dizygotic twins (0–10%), it appears that ASD is highly heritable (Abrahams & Geschwind, 2008). However, despite the high heritability there appears to be no single genetic cause for ASD.

The use of genome‐wide association studies, whole‐genome linkage studies, SNP analyses, and copy number variation screening have resulted in several candidate ASD genes (Abrahams & Geschwind, 2008). There are several single‐gene mutation syndrome disorders that are associated with an increased risk of developing ASD including Fragile X (*FMR1*), Rett Syndrome (*MECP2*), Tuberous sclerosis (either *TSC1* or *TSC2*), Timothy syndrome (*CACNA1C*), Angelman syndrome (*UBE3A*), and Cowden syndrome (*PTEN*) (Belmonte & Bourgeron, 2006; Butler et al., 2005; Nagarajan et al., 2008; Splawski et al., 2004; Wiznitzer, 2004). Despite the investigation of the underlying genes of ASD, only 10–20% of all identified genetic risk factors for the disorder have been uncovered (Abrahams & Geschwind, 2008). However, one common factor that has been observed in many of the single‐gene mutation syndrome disorders, such as Fragile X, Tuberous sclerosis, and Cowden syndrome, is hyperactivation of the PI3K/Akt/mTOR intracellular signaling pathway (Amir et al., 1999; Baker, Piven, & Sato, 1998; Hatton et al., 2006; Matsuura et al., 1997). Mutations of the suppressors of the pathway, *TSC1*,* TSC2*, or *PTEN* in mice, produce uncontrolled activation of the mTORC1 signaling cascade that results in macrocephaly and overgrowth in cellular and dendritic properties (Kwon et al., 2006; Meikle et al., 2008; Zeng, Xu, Gutmann, & Wong, 2008; Zhou et al., 2009). The Phosphoinositide 3‐kinase (PI3K) signaling cascade is activated by nutrients, growth factors and hormones, and is inhibited by PTEN (phosphatase and tensin homolog on chromosome 10).

Previous investigations in animal models of Fragile X, Tuberous sclerosis, and Cowden syndrome have reported ASD‐like behavioral deficits. Mice with deletion of *Fmr1* have deficits in social behavior, an increase in repetitive behavior, and communication deficits (Frankland et al., 2004; Gauducheau et al., 2017; Hodges, Nolan, Reynolds, & Lugo, 2017; Mineur, Huynh, & Crusio, 2006; Mineur, Sluyter, de Wit, Oostra, & Crusio, 2002). Mice with deletion of *Tsc1 or Tsc2* have similar ASD‐like deficits (Reith et al., 2013). For Cowden syndrome, which involves a mutation in the phosphatase *PTEN*, several conditional knockouts have been created. The neuronal subset‐specific KO for *Pten* (NS‐*Pten*) shows deficits in social behavior and aberrant repetitive behavior (Lugo et al., 2014). No deficits in ultrasonic vocalizations (USVs) have been reported (Lugo et al., 2014).

USVs are identified as “whistle‐like sounds” with frequencies ranging from 30 to 90 kHz (Branchi, Santucci, & Alleva, 2001). They can be emitted when a pup is separated from its parents, during social play, and are used during courtship and mating rituals, in addition to social investigation in adult mice (D'Amato, Scalera, Sarli, & Moles, 2005; Nyby, 1983; Panksepp et al., 2007). Research also suggests that altered USVs are an important component of the phenotype for various neurodevelopmental conditions, including ASD, Fragile X, and tuberous sclerosis complex (Reynolds, Nolan, Jefferson, & Lugo, 2016; Scattoni, Gandhy, Ricceri, & Crawley, 2008; Tsai et al., 2012).

The prior study that investigated USVs in NS‐*Pten* mice examined USVs using the Ultravox recording system which provides only the broad quantitative differences in calls, focusing on the amount of calls present at four specific frequencies (Lugo et al., 2014). For our study, we will use a full spectrum analysis recording system, which can allow for the investigator to analyze both quantitative and qualitative features of ultrasonic vocalizations, providing information regarding the amount of calls over a broad range, details about the temporal characteristics of the calls, and the different types of calls used. Since the full spectrum analysis program allows for a more in‐depth characterization of USVs, it is pragmatic to reassess the NS‐*Pten* model for any differences that may be detected on a more sensitive system. An additional focus of this paper will be to examine whether the changes in USVs are sex‐specific. In light of past evidence, we hypothesize that there would be no quantitative difference in vocalizations between WT and NS‐*Pten* KO mice. However, we also hypothesized that there would be qualitative differences present between the groups. We investigated this by eliciting vocalizations from KO and WT mice, including both males and females, on postnatal days (PD) 8 and 11 by way of the maternal isolation paradigm. Analyses of the USVs were conducted to examine both the spectral and temporal variability between WT and KO mice.

## 2 MATERIALS AND METHODS

### 2.1 Animals and housing

The NS‐*Pten* mice used were on a FVB‐based backcrossed background strain that had been bred for more than 10 generations and have been previously described (RRID:MGI:3714016) (Kwon et al., 2006). NS‐*Pten* ^*loxP*/+^ heterozygote parents were bred and used to produce NS‐*Pten* ^+/+^ wild‐type (WT), NS‐*Pten* ^*loxP*/+^ heterozygous (HT), and NS‐*Pten* ^*loxP*/loxP^KO pups. Pups were clipped on PD 7 and raised with their littermates, as well as both parents, in the home cage. All animals were retained throughout testing. A total of 33 litters, amounting to 54 NS‐*Pten* WT and KO pups, were used in this study. They comprised four groups: male WT (*n *=* *14), male KO (*n *=* *12), female WT (*n *=* *15), and female KO (*n *=* *13) mice, all of which were tested in the afternoon during the light cycle. The mice were generated and group housed at Baylor University in a room on a 12‐hr light/dark diurnal cycle held at 22⁰C, with the mice given *ad libitum* access to both food and water. All test procedures were carried out in compliance with the National Institute of Health Guidelines for the Care and Use of Laboratory Animals along with Baylor University's Institutional Animal Care and Use Committee.

### 2.2 Ultrasonic vocalizations

In this study USVs were elicited by a maternal isolation paradigm, as this has previously been shown to consistently initiate vocalizing behavior in pups (Shair, 2007). Mice were tested on both postnatal days 8 and 11 because vocalizations typically increase around PD 5, reach their peak around PD 7, and decrease around PD 14 (Branchi, Santucci, Vitale, & Alleva, 1998; Elwood & Keeling, 1982). Prior to testing, the pups were weighed and allowed to habituate in the testing room for 30 min. Next, they were removed from their dam in the room temperature home cage and the pups were put into a clean, preheated cage. We then individually tested each mouse by removing the mouse from the warmed cage and placing it in another 22°C cage, as detailed in numerous other studies (Scattoni et al., 2008; Shair, 2007; Tsai et al., 2012). The mouse was then placed within a 40 cm × 40 cm × 30 cm sound‐attenuated chamber. Their vocalizations were recorded for 2 min using an ultrasonic microphone (CM16/CMPA, Avisoft Bioacoustics, Germany, part #40011) and an ultrasound recording program (UltraSoundGate 116Hb, Avisoft Bioacoustics, part # 41161/41162). Following testing, the pups were placed back in the prewarmed cage. Once all mice were tested they were returned to their dam.

### 2.3 Ultrasonic vocalization analysis

The Avisoft SASLab Pro software (Avisoft SASLabPro, RRID:SCR_014438) was used to perform a Fast Fourier transformation (FFT) on the recorded data, creating a spectrogram with an FTT length of 1024, a time window overlap of 75%, with a 100% Hamming Window, a frequency resolution of 488 Hz, a sampling frequency of 22050, and a time resolution of 1 ms as detailed in Scattoni et al. (2008). The calls were visually identified using 1 of 10 distinct categories based on internal pitch changes as well as the length and shape of the individual calls. The categories were: complex, harmonic, two syllable, upward, downward, flat, chevron, short, composite, and frequency steps. Complex calls contained one syllable with two or more directional changes in pitch >6.25 kHz. Harmonic calls comprised a single call with additional calls of varying frequencies surrounding the primary call. Two syllable calls contained a flat or downward call with another short, punctuated call near the end. Both upward and downward call types have a continuous increase or decrease in pitch >12.5 kHz, ending with a frequency at least 6.25 kHz more or less than the initial pitch. Flat calls have a consistent beginning and ending of the call, with the pitch frequency deviating less than 3 kHz throughout the call's duration. Chevron calls have an “inverted‐U” shape, with a continuous increase in pitch that is >12.5 kHz immediately followed by a decrease in pitch >6.25 kHz. Short calls are less than 5 ms in length. Composite calls contain two harmonically independent components that are emitted at the same time. Frequency steps are calls with an abrupt frequency change that appears as a vertical discontinuous step but with no interruption of time (Scattoni et al., 2008). Additionally, the experimenter was blind to the condition of the animal at the time of scoring.

### 2.4 Behavioral data analysis and statistics

All statistical analyses were conducted using Graphpad Prism 7 software (La Jolla, CA) or SPSS 20.0 (IBM, USA). The differences in the quantity of calls between the groups and across both days were analyzed with a repeated‐measures ANOVA with genotype and sex as between‐subjects factors. A MANOVA was run to analyze the differences in the vocalization parameters. This was followed by independent *t*‐tests, grouped by genotype, or Mann‐Whitney *U* tests, when homogeneity of variance assumptions were violated, that detected the specific differences in mean duration, peak frequency, fundamental frequency, and peak amplitude, for each group over each day.

Call type was analyzed with a Pearson Chi‐Square, along with individual *z*‐tests, to compare significant call type proportions between genotypes, sexes, and postnatal days. A value of *p *<* *.05, was considered significant for each statistical test, with figures depicting the mean ± standard error of the mean (SEM).

## 3 RESULTS

### 3.1 Quantity of calls

**Figure 1:**
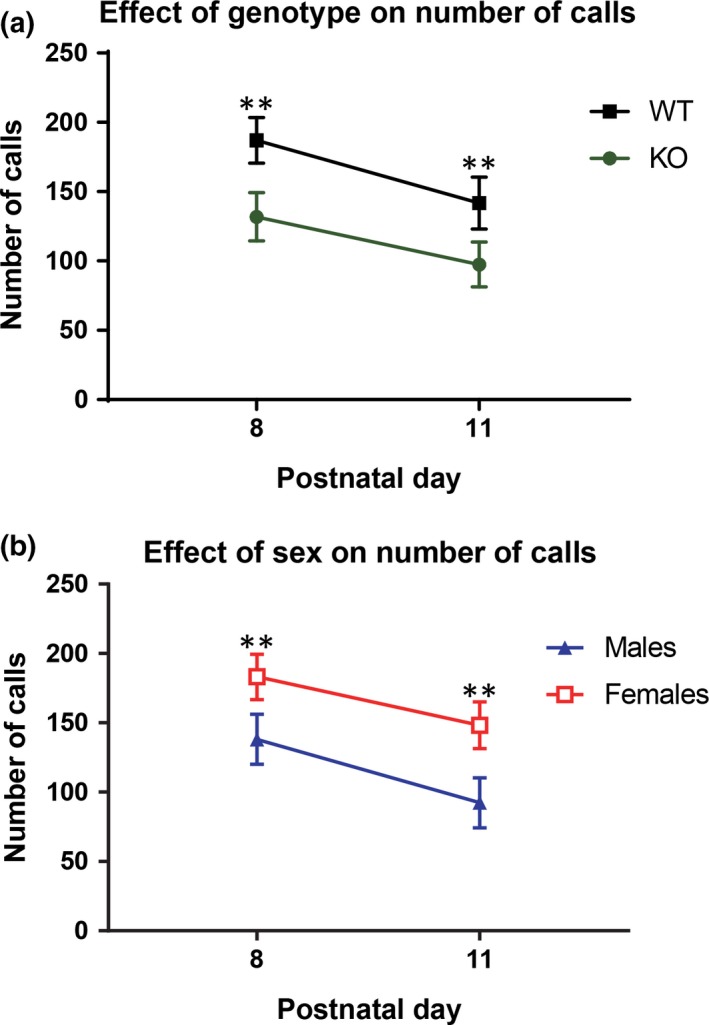
Effect of Genotype and Sex on the Quantity of Calls for PD 8 and PD 11. Mean quantity of calls for genotype and sex on PD 8 and PD 11. (a) WT mice emitted significantly more calls than KO mice, with more USVs being recorded on PD 8 than on PD 11. (b) Females emitted significantly more calls than males, with more USVs being recorded on PD 8 than on PD 11. The data points represent the mean and the error bars represent the standard error of the mean. WT male: *n *= 14, KO male *n* = 12, WT female: *n* = 15, KO female: *n* = 13. * = *p *<* *.05; ** = *p *<* *.01; *** = *p *<* *.001

An analysis of the mean number of calls emitted from each group across both days revealed a significant ANOVA with genotype and sex as the between subjects factors and the number of calls on PD 8 and the number of calls on PD 11 as the within subjects factors. There was a main effect for genotype (*F*_1,50_ = 7.06, *p *=* *.01), sex, (*F*_1,50_ = 6.96, *p *=* *.01), and for the amount of calls emitted on PD 8 when compared with PD 11, (*F*_1,50_ = 7.33, *p *=* *.01). KO mice emitted fewer vocalizations than WT mice and males emitted fewer vocalizations than females (Figure 1a,b). There was a significant reduction in the number of calls that were emitted by WT and KO mice on PD 11 compared with PD 8. Similarly, males and females also emitted fewer calls on PD 11 than on PD 8. There were no interactions found between genotype and sex (*F*_1,50_ = 2.6, *p *=* *0.11), between genotype over PD 8 and 11 (*F*_1,50_ = 0.16, *p *=* *0.70), sex over the 2 days (*F*_1,50 _= 0.1, *p *=* *0.76), or genotype by sex over time (*F*_1,50_ = 0.7, *p *=* *0.4).

### 3.2 Call type compositions

**Figure 2:**
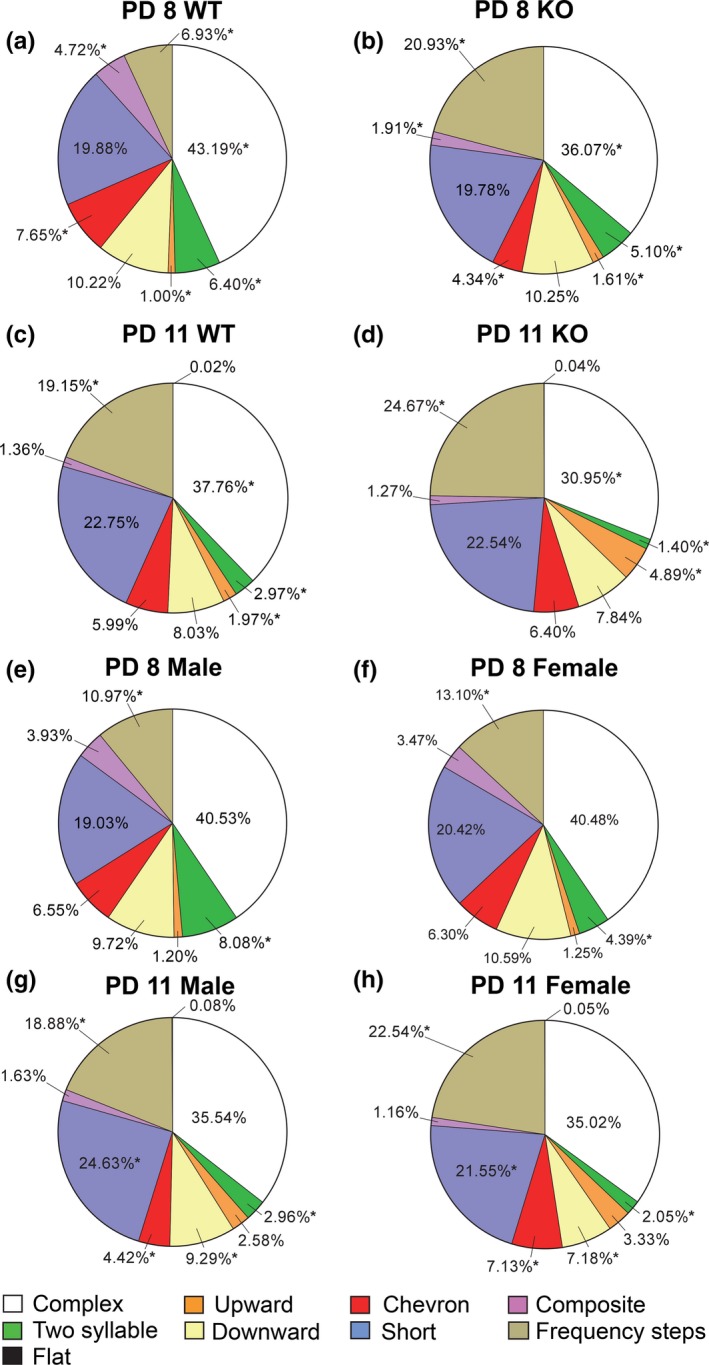
Call Type Composition per Genotype, Sex, and Day. (a) Call types for PD 8 WT mice. (b) Call types for PD 8 KO mice. (a,b) PD 8 KO animals emitted more frequency steps and upward calls, but fewer complex, two syllable, chevron, and composite calls when compared with WT mice. (c) Call types for PD 11 WT mice. (d) Call types for PD 11 KO mice. (c,d) PD 11 KO animals emitted more frequency steps and upward calls, but less complex and two syllable calls when compared with WT mice. (e) Call types for PD 8 males. (f) Call types for PD 8 females. (e,f) PD 8 males emitted more two syllable cries, but less frequency steps calls when compared with females. (g) Call types for PD 11 males. (h) Call types for PD 11 females. (g,h) PD 11 males emitted more two syllable, short, and downward call types, but fewer chevron and frequency steps calls when compared with females. * = *p *<* *.05; ** = *p *<* *.01; *** = *p *<* *.001

Even though there were significant genotype and sex differences, we wanted to examine the specific types of calls that were emitted to determine if certain calls are genotype or sex‐specific. When examining the spectral properties of call types, a Pearson Chi‐Square analysis revealed significant population differences between the composition of calls for WT and KO mice emitted on PD 8 (*X* ^*2*^(7, *N *= 8718) = 445.83, *p* < .001) and on PD 11 (*X* ^*2*^(8, *N *= 6549) = 101.43, *p* < .001). Proportional differences detected with z‐tests found that on PD 8 KO animals emitted a significantly greater amount of frequency steps and upward calls when compared with WT mice (*p *<* *.05) (Figure 2a,b). However, KO mice produced significantly less complex, two syllable, chevron, and composite calls (*p *<* *.05) (Figure 2a,b). On PD 11, KO animals showed a similar pattern, emitting significantly more frequency steps and upward calls (*p *<* *.05), with WT mice producing more complex and two syllable calls (*p *<* *.05) (Figure 2c,d).

Sex‐specific call type differences were also detected. A Pearson Chi‐Square analysis revealed significant population differences between male and female call types for both PD 8 (*X* ^*2*^(7, *N *= 8718) = 61.58, *p* < .001) and PD 11 (*X* ^*2*^(8, *N *= 6549) = 53.46, *p* < .001). Proportional differences detected with *z*‐tests found that on PD 8 males emitted significantly more two syllable cries (*p *<* *.05), whereas females produced more frequency steps calls (*p *<* *.05) (Figure 2e,f). On PD 11, males emitted more two syllable, short, and downward call types (*p *<* *.05), while females produced significantly more chevron and frequency steps calls (*p *<* *.05) (Figure 2g,h).

### 3.3 Spectral and temporal characteristics

We then analyzed the spectral and temporal characteristics of ultrasonic vocalizations for PD 8. Our analyses revealed a significant MANOVA for PD 8, with genotype and sex as the fixed factors and mean duration, peak frequency, fundamental frequency, and peak amplitude as dependent factors. There was a main effect for genotype (*F*_4,8711_ = 73.09, *p *<* *.001), sex (*F*_4,8711_ = 35.98, *p *<* *.001), and an interaction between genotype and sex (*F*_4,8711_ = 8.03, *p *<* *.001). We then examined the specific between‐subjects effects. There was a main effect of genotype for mean duration (*F*_1,8714_  = 24.1, *p *<* *0.001), peak amplitude (*F*_1,8714_ = 249.0, *p *<* *0.001), and peak frequency (*F*_1,8714_ = 49.4, *p *<* *0.001), but not for the mean fundamental frequency (*F*_1,8714 _= 1.13, *p* = 0.29). There were main effects of sex for the mean duration (*F*_1,8714_ = 12.9, *p *<* *0.001), fundamental frequency (*F*_1,8714_ = 5.4, *p *<* *0.05), peak amplitude (*F*_1,8714_ = 110.0, *p *<* *0.001), and peak frequency (*F*_1,8714_ = 43.9 *p *<* *0.001). There was an interaction between genotype and sex for mean duration (*F*_1,8714_ = 9.5, *p *<* *0.01), fundamental frequency (*F*_1,8714_ = 6.7, *p *<* *0.05), and peak amplitude (*F*_1,8714_ = 10.8, *p *<* *0.001), but not for the mean peak frequency (*F*_1,8714_ = 0.001, *p* = 0.97).

**Figure 3:**
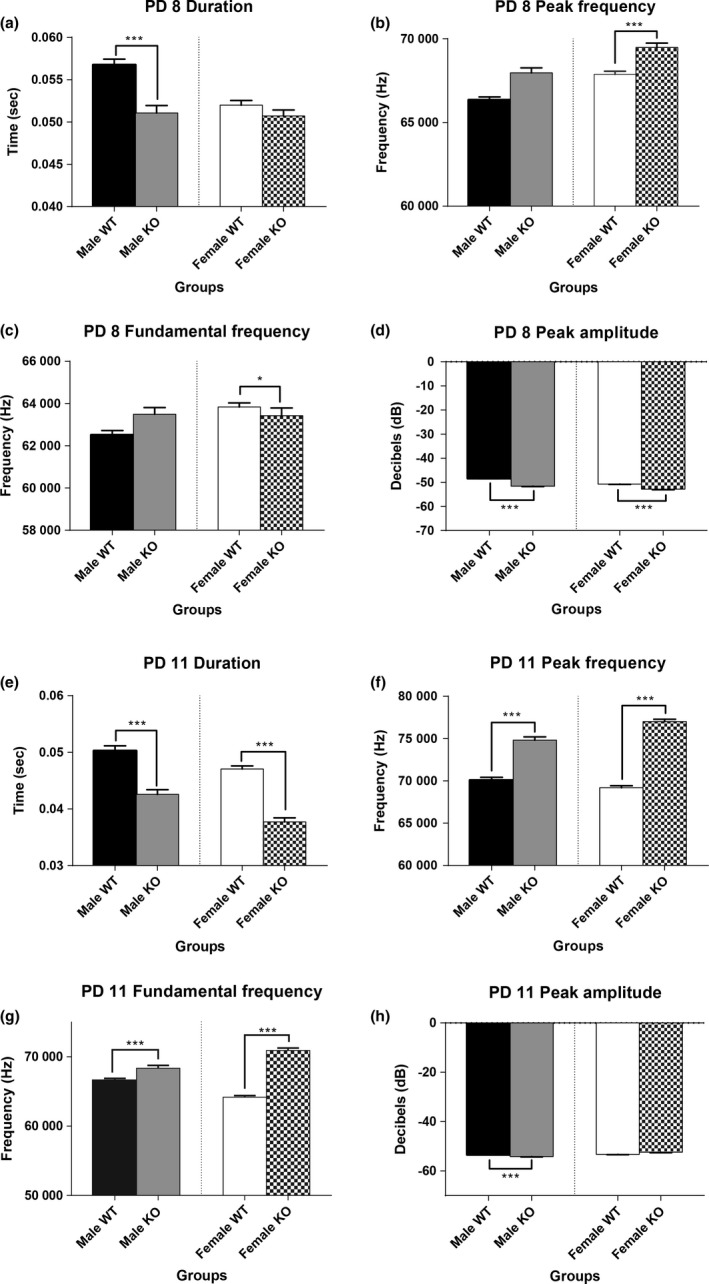
Spectral Characteristics of the Vocalizations. Mean duration, peak frequency, fundamental frequency, and peak amplitude for WT and KO mice on PD 8 and PD 11. Male KO mice on PD 8 emitted calls of a shorter mean duration (a), and a lower mean peak amplitude (d) than male WT mice. Female KO mice on PD 8 emitted calls at a higher mean peak frequency (b), a lower fundamental frequency (c), and a lower mean peak amplitude (d) than WT females. Male KO mice on PD 11 emitted USVs of a shorter mean duration (e), higher mean peak frequency (f), higher mean fundamental frequency (g), and a lower mean peak amplitude (h) than WT males. Female KO mice on PD 11 emitted calls of a shorter mean duration (e), higher mean peak frequency (f), and a higher mean fundamental frequency (g) than WT females. The bars represent the mean and the error bars represent the standard error of the mean. * = *p *<* *.05; ** = *p *<* *.01; *** = *p *<* *.001

Due to the interaction of group and sex we performed independent *t*‐tests or nonparametric Mann‐Whitney *U* tests between WT and KO mice per sex. Significant differences between WT and KO mice on PD 8 were detected, with KO males emitting calls of a shorter average duration (*t*_3588_ = 5.08, *p *<* *.001) (Figure 3a) and lower mean peak amplitude (*U* = 1217712, *p *<* *.001), than WT males (Figure 3d). Female KO mice on PD 8 displayed calls with a higher mean peak frequency (*U* = 2853297, *p *<* *.001) (Figure 3b), a lower mean fundamental frequency (*U* = 2948392, *p* = .04) (Figure 3c), and a lower mean peak amplitude (*U* = 2386907, *p *<* *.001) than WT females (Figure 3d).

Analyses of the spectral and temporal characteristics of ultrasonic vocalizations revealed a significant MANOVA for PD 11 with genotype and sex as the fixed factors and mean duration, peak frequency, fundamental frequency, and peak amplitude as dependent factors. There was a main effect for genotype (*F*_4,6542_ = 151.55, *p *<* *.001), sex (*F*_4,6542_ = 37.18, *p *<* *.001), and an interaction between genotype and sex (*F*_4,6542_ = 22.88, *p *<* *.001). The between subjects analyses revealed a main effect of genotype for mean duration (*F*_1,6545_ = 123.8, *p *<* *0.001), fundamental frequency (*F*_1,6545_ = 157.7, *p *<* *0.001), and peak frequency (*F*_1,6545_ = 408.7, *p *<* *0.001), but not for mean peak amplitude (*F*_1,6545_ = 0.007, *p* = 0.93). There was a main effect of sex for mean duration (*F*_1,6545_ = 27.8, *p *<* *0.001), peak amplitude (*F*_1,6545_ = 32.1, *p *<* *0.001), and peak frequency (*F*_1,6545_ = 4.3, *p *<* *0.05), but not for the mean fundamental frequency (*F*_1,6545_ = 0.1, *p* = 0.76). There were several statistically significant interactions found between genotype and sex. The differences were found in the mean fundamental frequency (*F*_1,6545_ = 52.9, *p *<* *0.001), peak amplitude (*F*_1,6545_ = 28.9, *p *<* *0.001), and peak frequency (*F*_1,6545_ = 24.3, *p *<* *0.001), but not for the mean duration (*F*_1,6545_ = 1.2, *p* = 0.27).

When examining males on PD 11, KO mice emitted calls of an average shorter duration (*U* = 625954, *p *<* *.001) (Figure 3e), higher mean peak frequency (*t*_2397_ = 9.33, *p *<* *.001) (Figure 3f), higher mean fundamental frequency (*U* = 658154, *p *<* *.001) (Figure 3g), and lower mean peak amplitude (*U* = 676598, *p *<* *.001) than WT males (Figure 3h). Similarly, when analyzing females on PD 11, KO mice emitted cries of a shorter mean duration (*U* = 1487283, *p *<* *.001) (Figure 3e), higher mean peak frequency (*U* = 1161870, *p *<* *.001) (Figure 3f), and a higher mean fundamental frequency (*U* = 1305243, *p *<* *.001) than WT females (Figure 3g).

## 4 DISCUSSION

In this study, we compared the quantity, call types, and spectral characteristics of neonatal ultrasonic vocalizations in WT and NS‐*Pten* KO male and female pups. We found that NS‐*Pten* KO mice emitted significantly fewer vocalizations than WT mice. Additionally, male pups emitted fewer vocalizations than females on PD 8 and 11. Overall, there was a general reduction in the number of calls from PD 8 to PD 11. The types of calls emitted were also different between WT and KO mice. Lastly, significant differences in the acoustic and temporal structures of the calls were revealed. Specifically, NS‐*Pten* KO males on PD 8 emitted calls with a shorter mean duration and lower mean peak amplitude. NS‐*Pten* KO females had calls of a higher mean peak frequency, lower fundamental frequency, and a lower peak amplitude. On PD 11, NS‐*Pten* KO males emitted calls with a shorter mean duration, higher peak frequency, higher fundamental frequency, and a lower mean peak amplitude, whereas NS‐*Pten* KO females emitted calls of a shorter mean duration, higher peak frequency, and a higher mean fundamental frequency.

Perhaps, the most important finding in this study was the aberrant vocalizations seen in NS‐*Pten* KO mice. Similarly deviant vocalizations have also been observed in *Fmr1‐* and *Tsc1‐*deficient pups, therefore our results contribute to a consistency of communication deficits found across several mTOR models (Reynolds et al., 2016; Tsai et al., 2012; Young, Schenk, Yang, Jan, & Jan, 2010). Specifically, *Fmr1* KO pups were shown to emit significantly fewer vocalizations than their WT counterparts when separated from their mother (Reynolds et al., 2016). In contrast, *Tsc1* HT pups emitted significantly more vocalizations than their controls (Tsai et al., 2012). Thus, while the particular quantity of vocalizations emitted is not always constant across different mTOR models, the underlying aberrant vocalizing patterns are still observed, demonstrating a consistency between our findings and those in related studies.

Another congruous finding between our results and those seen in another mTOR model is the average length of the vocalization emitted. Both male and female *Fmr1* KO pups have been shown to exhibit a decreased call duration when compared with wild types, resembling our findings in this study (Reynolds et al., 2016). Call duration is significant as dams have been shown to prefer a longer call over a shorter one (Smith, 1976). Dams have even been shown to not respond to vocalizations lasting under 30 ms (Ehret, 1992). Taken together, this indicates that mTOR dysfunction may put the corresponding KO pups at a disadvantage in eliciting their dams’ response due to their decreased call length.

While the results garnered from our study do appear to fit in well with the literature as a whole, there is one notable exception, as the current findings are contrary to a similar study conducted previously in our lab. Specifically, a prior study from our lab reported no difference in the quantity of vocalizations for NS‐*Pten*‐KO mice on PD 10 and 12 (Lugo et al., 2014). This discrepancy is perhaps best explained by the different software programs used to record the USVs. Whereas the current study utilized a full spectrum analysis program, Lugo et al. (2014) employed the Ultravox system. The full spectrum analysis equipment uses a broad‐spectrum microphone that is able to identify frequencies ranging from 0 to 125 kHz, allowing for the detection of all possible neonatal cries (Avisoft Bioacoustics). Conversely, the Ultravox system utilizes microphones set to a specific frequency; for the study in question they were set to 40, 50, 60, and 70 kHz (Lugo et al., 2014). The detectors are designed to pick up calls within 1 kHz around the frequency that the detector is set to. Therefore, approximately 8 kHz between each detector could be missed and calls lower than 39 and greater than 81 would not be detected. As mice are able to vocalize anywhere between 30 to 90 kHz, it is quite possible that some of the calls were simply not detected using the Ultravox system. Additionally, the full spectrum analysis program allows the experimenter to delete background noise that may have been incorrectly detected as a call; no such option exists for the Ultravox system. While no study has directly compared the vocalizations recorded in the Ultravox program to the Avisoft program, the numerous differences underscoring these two systems form the most likely explanation for any variation present in the results.

While the current vocalization literature is promising, future studies could look more closely at the USV patterns of females in various models, as this presents an area that is currently underrepresented. Future studies could also examine other characteristics of calls in mTOR models such as the average peak amplitude, peak frequency, or fundamental frequency, to provide this study's findings more context. Additionally, measures of maternal behavior could be investigated. Specifically, maternal retrieval tests, wherein the dam would choose between the recordings of WT or KO USVs, could provide a valuable insight into the relationship between altered neonatal cries and the corresponding maternal response. Studies could also add in more time points to see if the developmental trajectory of call frequency is altered in NS‐*Pten* KO mice. Lastly, studies could investigate drug‐based interventions to see if the atypical vocalization behavior seen in mTOR models normalizes in the presence of a treatment.

Overall, our results demonstrate a striking consistency in aberrant ultrasonic vocalizations that is seen across numerous mTOR models. Furthermore, there is evidence to suggest that the temporal characteristic of the calls, their average duration, is also uniformly impacted. In conclusion, through examining an underrepresented model, this study addressed a significant deficit in the mTOR vocalization literature. Additionally, it not only provides context for findings in the *Fmr1* and *Tsc1* models, but also further implicates the mTOR pathway as playing a potential causative role in aberrant vocalizations in neonates. Perhaps most importantly, this study significantly contributes to the characterization of a molecular mechanism responsible for an autistic‐like phenotype, thereby partially elucidating the underlying disorder. Thus, as our study helps to facilitate a better understanding of the NS‐*Pten* phenotype it is, by proxy, also integral to garnering a better understanding of autism.

## DISCLOSURES

Neither of the authors have any conflicts of interest to declare.
